# Myelin plasticity and behaviour — connecting the dots

**DOI:** 10.1016/j.conb.2017.09.014

**Published:** 2017-12

**Authors:** Malte Sebastian Kaller, Alberto Lazari, Cristina Blanco-Duque, Cassandra Sampaio-Baptista, Heidi Johansen-Berg

**Affiliations:** Nuffield Department of Clinical Neurosciences, John Radcliffe Hospital, University of Oxford, Oxford OX1 2JD, United Kingdom

## Abstract

Myelin sheaths in the vertebrate nervous system enable faster impulse propagation, while myelinating glia provide vital support to axons. Once considered a static insulator, converging evidence now suggests that myelin in the central nervous system can be dynamically regulated by neuronal activity and continues to participate in nervous system plasticity beyond development. While the link between experience and myelination gains increased recognition, it is still unclear what role such adaptive myelination plays in facilitating and shaping behaviour. Additionally, fundamental mechanisms and principles underlying myelin remodelling remain poorly understood. In this review, we will discuss new insights into the link between myelin plasticity and behaviour, as well as mechanistic aspects of myelin remodelling that may help to elucidate this intriguing process.

## Highlights

• Changes in white matter and myelin are associated with learning during adulthood across species.
• The causal link between myelin plasticity and behaviour remains elusive.
• Preventing the differentiation of new OLs can impair learning within the first few hours.
• Myelin remodelling may occur through many different routes and mechanism.
• The functional arrangement of myelination along axons can be complex and diverse.

**Current Opinion in Neurobiology** 2017, **47**:86–92

This review comes from a themed issue on **Glial biology**

Edited by **Alison Lloyd** and **Beth Stevens**

For a complete overview see the Issue and the Editorial

Available online 17th October 2017

**http://dx.doi.org/10.1016/j.conb.2017.09.014**

0959-4388/© 2017 The Authors. Published by Elsevier Ltd. This is an open access article under the CC BY license (http://creativecommons.org/licenses/by/4.0/).

## Introduction

The acquisition of myelinating glia was a critical evolutionary advancement that enabled the development of increasingly complex nervous systems. In the central nervous system (CNS) glia cells called oligodendrocytes (OLs) form myelin sheaths (also called internodes) by wrapping long segments of axons with a multi-layered sheath of extended cell membrane. Myelination was first understood to enable faster impulse propagation in axons more than 60 years ago, yet it took a long time to recognise the mechanistic complexity of this cellular process and the diverse roles that myelination plays in the formation and functioning of the vertebrate nervous system [1].

White matter (WM) gets its colour from the abundance of myelinated axons in the connective tracts of the CNS, which make up about half of the human brain volume. The past few years have seen a refreshed interest in WM as a driver of behaviour, as converging evidence has indicated that myelin in the CNS can be dynamically regulated by neuronal activity and experience [2, 3, 4]. Although this phenomenon has been recognised in cell culture studies for some time [5, 6], its demonstration *in vivo*, and its potential contribution to behaviour, has been shown only recently [7••, 8••]. Indeed, converging evidence from a number of different experimental approaches, including neuroimaging studies in humans [9, 10•, 11] and rodents [12], genetically modified mice [7^••^] as well as manipulation of neural activity in both rodents and zebrafish [13, 14, 15], suggest an active role for dynamic myelination in adult brain plasticity and indicate myelin plasticity may be an additional route by which experience can shape brain structure and function.

**Figure 1:**
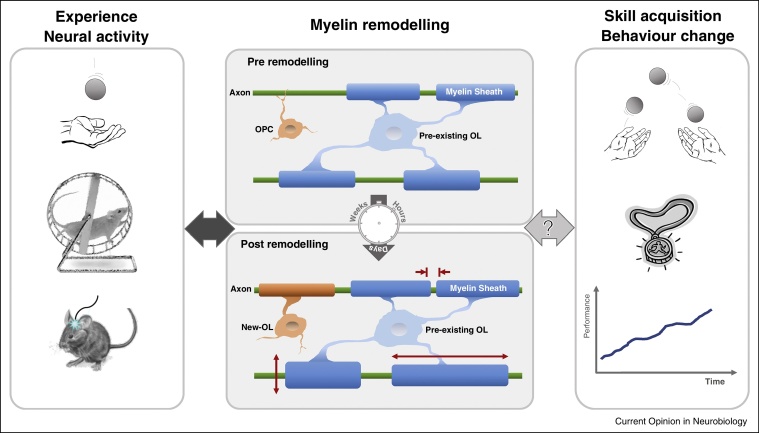
Myelination can be dynamically remodelled by neural activity and experience even during adulthood, yet its role in learning remains elusive. Experiences such as learning to juggle [9] or playing a computer game [30] are associated with structural changes in white matter pathways in humans, while learning a motor skill can lead to changes in myelination in rodents [12]. Indeed, neural activity can regulate changes in myelin-forming cells within an active circuit, as demonstrated by *in vivo* optogenetic techniques in awake, behaving mice [13]. Such myelin plasticity may occur through many different routes (see Figure 2). While changes in myelination have mainly been investigated after days or weeks, recent evidence suggests an active requirement of New-OL within the first hours of skill acquisition [8^••^]. However, if and to what extent adaptive changes in myelination can facilitate behavioural change and skill acquisition remains poorly understood. Learning more about the underlying biological mechanism, such as the speed at which experience can be translated into adaptive changes in myelination, will be critical to understand the role that myelin plasticity plays in the nervous system.

Our current understanding of brain plasticity is mainly centred around the concept of synaptic plasticity, which is supported by widely acknowledged theoretical frameworks, decades of experimental work and neural modelling [16]. In contrast, evidence for the plasticity of myelin has only started to converge in recent years and consequently its functional significance and underlying mechanisms remain undefined. Hence, it is still unclear how myelination can be dynamically regulated by experience to facilitate adaptive changes in neural network behaviour that underlie behavioural change (Figure 1).

## Association between behaviour and WM during development and ageing

Correlated changes in WM and cognitive functions over the human lifespan offer a link between myelin and cognitive and sensorimotor development. For instance, maturation of sensorimotor or language-related tracts are associated with the development of these basic skills in childhood [17, 18, 19], whereas maturation of fronto-parietal [18, 20, 21] and fronto-striatal [22, 23, 24] WM pathways correlates with protracted development of executive functions and behavioural control during adolescence and early adulthood. At the other end of the lifespan, ageing is accompanied by axon and myelin deformation and degeneration even in the absence of specific pathological conditions, as indicated by post-mortem histological studies [25, 26]. Mirroring the observed loss of myelinated fibres, changes in WM diffusion metrics are reliably associated with ageing [27]. Interestingly, ageing seems to predominantly affect a network of higher-order brain regions that myelinate relatively late during adolescence and seem especially vulnerable to disorders during early and late development [28]. Accumulating evidence indicates that such age-related declines in cerebral WM integrity may contribute to cognitive deficits associated with ageing [29], although this relationship remains a subject of debate. However, while developmental and age-related WM changes indicate an important association between myelin and healthy brain function, such associations are hindered by a wide range of concomitant lifespan-related biological changes and can provide little mechanistic insight into whether or how myelin can shape behaviour.

## Myelin plasticity may illuminate the link between myelin and behaviour

**Figure 2:**
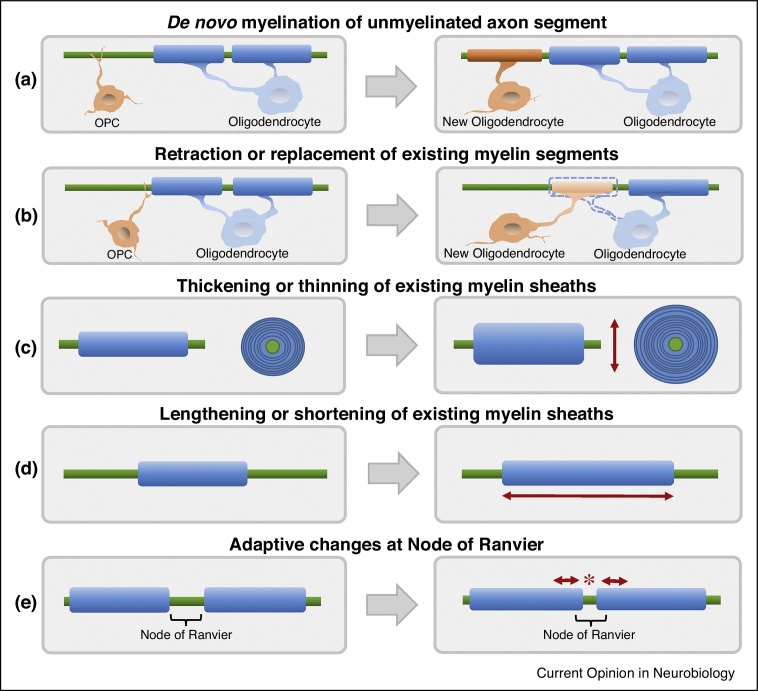
Different cellular processes may lead to dynamic changes in myelination during adulthood. Axons (green) in the CNS can be wrapped by myelin segments (blue), which are formed by oligodendrocytes (OLs) during development. Converging evidence indicates that myelination can be dynamically remodelled by activity-dependent and experience-dependant mechanism, even during adulthood. **(a)** Oligodendrocyte precursor cells (OPCs, orange) are an abundant proliferating cell population in the adult CNS and have the potential to differentiate into new myelinating oligodendrocytes [36]. Such *de novo* myelination (orange segment) can occur at previously unmyelinated segments, or **(b)** can replace retracting or damaged myelin segments of pre-existing OLs. **(c,d)** Additionally, pre-existing oligodendrocytes may also adjust structural parameters of their myelin sheath to modify nerve conduction velocity. Such myelin remodelling can be achieved by (c) altering the thickness of myelin segments though the addition or removal of membrane layers [38^•^], or (d) varying the length of myelin segments [44^•^]. Yet, it remains unclear if and to what extent myelin remodelling in the adult CNS can be mediated by pre-existing OLs. **(e)** Additionally, adjustment of node of Ranvier length has been suggested as another potential mechanism for tuning the arrival time of information in the CNS [46^•^]. While all of these mechanisms have the potential to change the information flow within neural networks, their relative contribution to adaptive myelin plasticity, as well as their mechanistic complexity, remain poorly understood.

While brain plasticity has mainly been studied in the context of activity-dependent changes at the synapse, converging evidence from animal and human studies now indicates that similar mechanisms can also regulate myelination. This previously overlooked phenomenon, termed myelin plasticity, may provide a complementary route through which experience shapes the brain [3]. Indeed, in healthy adults, evidence for a link between changes in WM structure and skill learning in humans is accumulating [9, 30, 31]. Using myelin-sensitive imaging methods [32, 33], a recent study found experience-dependent changes in myelin that were associated with changes in skilled movements in healthy young adults [10^•^]. However, in most studies, concurrent changes in other WM cells as well as in grey matter make it challenging to infer the functional relevance of myelin plasticity for any associated behavioural change. Allowing the possibility for more direct experimental interventions, rodent studies can provide important insights into the link between changes in myelin and behaviour [7••, 12, 13, 34], although potential differences in oligodendroglia dynamics between humans and other animals have to be considered [35, 36]. Conditional knock-out of the gene encoding the transcription factor MYRF (Myelin Regulatory Factor) in adult mice has provided the only evidence for a direct causal link between experience-dependent myelin plasticity and skill acquisition [7••, 8••]. By preventing the maturation of newly generated OLs in adult mice (Figure 2a,b), this knock-out seems to interfere with the mice's ability to master running on a complex wheel with irregularly spaced rungs [7••, 8••]. Importantly, if the mice had been exposed to the complex wheels prior to the genetic manipulation, their performance was unimpaired, suggesting that newly forming OLs play a specific role in acquisition of a novel skill. However, it remains to be determined whether these findings translate to other forms of learning and precisely which aspects of the behaviour are impaired. Using a different transgenic approach, another group indicated that the continuous generation of new OLs in the adult brain of mice may play a role in the maintenance of nodal integrity, and consequently, in the maintenance of motor functions, yet the study did not investigate motor learning [37].

Alongside myelin formed by newly differentiating OLs, remodelling of the existing myelin sheath represents another putative mechanism by which experience can shape brain structure (Figure 2c,d). Yet it remains unclear if and to what extent myelin remodelling in the adult CNS can be mediated by pre-existing OLs. To address this, a recent study demonstrated that conditional upregulation of cellular signalling pathways in pre-existing OLs of adult mice was sufficient to induce a subtle increase in myelination in the CNS *that was mainly driven by the addition* of new myelin to existing sheaths, rather than *de novo* myelination of naked axons [38^•^]. Additionally, a concurrent increase in nerve conduction velocity indicated that the observed myelin remodelling resulted in functional changes. To probe whether such upregulation of myelin gives incremental benefits or rather disturbs homeostatic fine-tuning, the authors assessed a variety of behaviours and only found evidence for slightly enhanced hippocampal-dependent emotional learning; motor learning and object recognition remained unaffected. While this study supports the notion that myelin remodelling in pre-existing myelin sheaths is a probable mechanism for myelin plasticity [39, 40], these findings also suggest that subtle and globally induced hypermyelination during adulthood does not lead to general cognitive or motor behavioural change. This could mean that behavioural performances is not consistently sensitive to subtle changes in myelination during adulthood. However, it is also possible that the CNS can compensate for such artificially induced hypermyelination, mechanisms of adaptive myelination related to learning remain largely unaffected by the genetic manipulation, and/or the influence of myelin remodelling on behaviour depends on the specific demands of the behaviour in question. Overall, these considerations are an important reminder that the link between myelin remodelling and behavioural adaptation remains poorly understood (Figure 1).

## Driver of behavioural change: myelin remodelling and conduction speed

Considering myelin's traditional function in modulating nerve conduction velocity, myelin plasticity may play a vital role in facilitating the flow and integration of information [41]. Additionally, precise timing of nerve impulses is essential for certain forms of synaptic plasticity [2, 42] and regulation of oscillatory neuronal activity in large and complex neural networks [43]. However, it has been methodologically and experimentally challenging to study how experience can influence nerve conduction velocity. Etxeberria and colleagues demonstrated that visual deprivation via monocular eyelid suture can lead to shortening of myelin sheath length in the respective optic nerve, which in turn reduced nerve conduction velocity [44^•^]. Additionally, genetically induced increases in CNS myelin sheath thickness have been linked to changes in nerve conduction speed and subtle behavioural effects [38^•^]. Recently, changes in the nodes of Ranvier have been considered as another mechanism by which experience can modulate nerve conduction speed [45, 46•], as altering the length of a node can change the node capacitance and the axial resistance for current flow into the internode (Figure 2e). Modelling of conduction speed based on nodal lengths imaged in the mouse CNS has shown that variations in the length of nodes can change the conduction velocity by about 20%, which is comparable to the changes produced by alterations in myelin sheath thickness or length [46^•^]. Interestingly, the change in membrane area needed to achieve a given change of conduction speed was estimated to be >270-fold smaller for a change in nodal length than for an alteration of the myelin sheath thickness or length, making it a potentially faster and more energy efficient mechanism.

Furthermore, it is noticeable that structural parameters of myelinated axons can deviate significantly from the canonically assumed relationship between axon dimeter and myelin thickness [47]. While investigating parts of the auditory system responsible for sound localisation, Ford and colleagues observed that structural parameters of myelin sheaths and nodes can differ significantly between different axons that compute different sound frequencies, and can even vary along a single axon [45]. Importantly, the authors claim, backed by simulations and *in vivo* and *in vitro* recordings, that such structural arrangements of myelination can play a significant role in information processing in the investigated network. While these observations describe myelination established during development, such findings indicate that the relationship between the myelin sheath and conduction velocity may be more complex than previously assumed, which will need to be considered when examining the functional significance of myelin remodelling and its role in the nervous system.

## Understanding the mechanisms underlying myelin plasticity will be essential to understanding its relevance for behaviour

Understanding the biological mechanisms underlying adaptive myelination is a key issue surrounding myelin plasticity, and one that will likely require intense translational efforts across disciplines, from molecular biology to neuroimaging [48]. A good example of this is the question of how fast myelin can be dynamically modulated by experience, which in turn has vast implications for its potential role in behavioural adaptation. Changes in WM microstructure, as assessed by MRI, have been detected after just 2 hours of playing a video game [30]. However, MRI metrics do not provide a direct measure of myelin. Hence, it is unclear if and to what extent myelin remodelling could contribute to such a rapid signal change, as most animal studies have investigated experience-dependent or activity-dependent changes in myelination in the timeframe of days or weeks [7••, 44•]. A recent experiment, however, indicated a causal link between behaviour changes and OL dynamics within a few hours. By identifying a novel marker of early OL differentiation (*Enpp6*), Xiao and colleagues were able to track OL differentiation in adult mice to show that maturation of newly forming OLs was increased within the first 2.5 hours of exposure to a complex running wheel [8^••^]. Interestingly, preventing the differentiation of new OLs caused a deficit in running performance in the same 2–3 hours time window, suggesting a very early and active requirement for newly differentiating OLs in learning.

Understanding the speed at which myelin can be remodelled may be important for unraveling its function in the nervous system. Myelin remodelling could be understood as a slower, secondary process that reacts to changes in the dynamics of neural networks. The purpose of such secondary plasticity could be the consolidation of learning by means of adjusting myelination patterns to the new needs of the neural network, as well as providing important homeostatic control. The evidence presented by Xiao and colleagues suggests that myelin plasticity can act in a similar temporal window as fast, primary plastic changes provided by synaptic plasticity. This suggests that these two mechanisms may interact more synergistically than previously thought. However, it remains unclear how such rapid changes in conduction velocity can be achieved. A subpopulation of oligodendrocyte precursor cells (OPCs) may be primed to rapidly respond to novel experience by differentiating into myelinating OLs [8^••^]. Such new OLs could establish the location of their new myelin segments within a few hours, as indicated by *in vitro* and *in vivo* zebrafish models [49, 50]. Nevertheless, it is unclear how such early processes can lead to significant alteration in conduction velocity. While it is possible that just one or a few wraps of new myelin might have beneficial effects, newly forming pre-myelinating OLs may also influence neural activity independent of *de novo* myelination — for example, through clustering of nodal components at ‘pre-nodes’, which can be induced by OL-secreted factors [51], or early metabolic coupling between newly differentiating OLs and axons [52]. Additionally, remodelling of existing myelin sheaths [38^•^], as well as adjustment of nodes of Ranvier length [45, 46•] provide alternative and fast routes by which myelin could adapt to neuronal activity changes. However, neither the relative contribution, nor the temporal dimensions of these mechanisms have been investigated.

Furthermore, it is still unclear if myelin plasticity is bi-directional. The vast majority of work in this area has investigated whether increasing activity results in increased number, length or thickness of myelin internodes. However, given the requirements for homeostasis in any biological system, it is plausible that a reciprocal phenomenon occurs, whereby certain types of activity could result in downregulation of myelin, analogous to long-term depression at the synapse. While social [53, 54] or visual deprivation [44^•^] have been associated with a reduction in the number of internodes or the number of myelin wraps that develop, there is currently no *in vivo* evidence for an activity-dependent mechanism that leads to a decrease in pre-existing myelination.

Finally, converging evidence suggests that oligodendroglia in the CNS are more heterogeneous than previously thought [55, 56], with potential variations of their intrinsic myelination capacity [57^•^]. Additionally, neural identity can influence respective profiles of myelination in mice [58], as well as activity-dependant myelination in the developing zebrafish [59^•^]. These findings indicate that the identity of neurons and OLs, as well as their idiosyncratic interactions, can determine the structural parameters and plasticity of their respective myelination. While this complicates the task of unravelling the functional role of myelin plasticity, it may simultaneously be an indicator of its significance.

## Concluding remarks

While the link between behaviour, myelin integrity and developmental myelination had been recognised in the past, the plastic regulation of myelination, even during adulthood, has gained increased recognition as an additional mechanism by which experience can shape brain structure. However, the role of adaptive myelination in facilitating and shaping behavioural change remains elusive and fundamental mechanisms underlying myelin remodelling are still poorly understood, and appear to be complex and diverse. Such complexity will need to be considered when trying to detect functionally relevant changes in myelination and to establish a conclusive link between myelin remodelling and behavioural change, which remains a key challenge in the field. Technological advances, such as electron microscopic volumetric reconstruction of brain tissue [60, 61] and models to translate between MRI signals and microscopy [62, 63], present promising tools to relate evidence across the different experimental approaches and to address the many unanswered questions regarding the role myelin plasticity might play in the nervous system.

## Funding information

MSK, AL and CDB are funded by PhD Studentships from the Wellcome Trust (Grant Numbers: 102393/Z/13/Z, 109062/Z/15/Z and 109059/Z/15/Z). HJB is funded by a Principal Research Fellowship from the Wellcome Trust (110027/Z/15/Z).

## Conflict of interest statement

Nothing declared.

## References

[1] Nave K-A. (2010). Myelination and support of axonal integrity by glia. Nature.

[2] Chang K-J., Redmond S.A., Chan J.R. (2016). Remodeling myelination: implications for mechanisms of neural plasticity. Nat Neurosci.

[3] Fields R.D. (2015). A new mechanism of nervous system plasticity: activity-dependent myelination. Nat Rev Neurosci.

[4] Purger D., Gibson E.M., Monje M. (2016). Myelin plasticity in the central nervous system. Neuropharmacology.

[5] Demerens C., Stankoff B., Logak M., Anglade P., Allinquant B., Couraud F., Zalc B., Lubetzki C. (1996). Induction of myelination in the central nervous system by electrical activity. Proc Natl Acad Sci U S A.

[6] Stevens B., Tanner S., Fields R.D. (1998). Control of myelination by specific patterns of neural impulses. J Neurosci.

[7••] McKenzie I.A., Ohayon D., Li H., Faria J.P., de Emery B., Tohyama K., Richardson W.D. (2014). Motor skill learning requires active central myelination. Science.

[8••] Xiao L., Ohayon D., McKenzie I.A., Sinclair-Wilson A., Wright J.L., Fudge A.D., Emery B., Li H., Richardson W.D. (2016). Rapid production of new oligodendrocytes is required in the earliest stages of motor-skill learning. Nat Neurosci.

[9] Scholz J., Klein M.C., Behrens T.E.J., Johansen-Berg H. (2009). Training induces changes in white matter architecture. Nat Neurosci.

[10•] Lakhani B., Borich M.R., Jackson J.N., Wadden K.P., Peters S., Villamayor A., MacKay A.L., Vavasour I.M., Rauscher A., Boyd L.A. (2016). Motor skill acquisition promotes human brain myelin plasticity. Neural Plast.

[11] Caeyenberghs K., Metzler-Baddeley C., Foley S., Jones D.K. (2016). Dynamics of the human structural connectome underlying working memory training. J Neurosci.

[12] Sampaio-Baptista C., Khrapitchev A.A., Foxley S., Schlagheck T., Scholz J., Jbabdi S., DeLuca G.C., Miller K.L., Taylor A., Thomas N. (2013). Motor skill learning induces changes in white matter microstructure and myelination. J Neurosci.

[13] Gibson E.M., Purger D., Mount C.W., Goldstein A.K., Lin G.L., Wood L.S., Inema I., Miller S.E., Bieri G., Zuchero J.B. (2014). Neuronal activity promotes oligodendrogenesis and adaptive myelination in the mammalian brain. Science.

[14] Mensch S., Baraban M., Almeida R., Czopka T., Ausborn J., El Manira A., Lyons D.A. (2015). Synaptic vesicle release regulates myelin sheath number of individual oligodendrocytes in vivo. Nat Neurosci.

[15] Hines J.H., Ravanelli A.M., Schwindt R., Scott E.K., Appel B. (2015). Neuronal activity biases axon selection for myelination in vivo. Nat Neurosci.

[16] Kreutz M.R., Sala C. (2012). Synaptic Plasticity: Dynamics, Development and Disease.

[17] Fields R.D. (2008). White matter in learning, cognition and psychiatric disorders. Trends Neurosci.

[18] Nagy Z., Westerberg H., Klingberg T. (2004). Maturation of white matter is associated with the development of cognitive functions during childhood. J Cogn Neurosci.

[19] Simmonds D.J., Hallquist M.N., Asato M., Luna B. (2014). Developmental stages and sex differences of white matter and behavioral development through adolescence: a longitudinal diffusion tensor imaging (DTI) study. NeuroImage.

[20] Mabbott D.J., Noseworthy M., Bouffet E., Laughlin S., Rockel C. (2006). White matter growth as a mechanism of cognitive development in children. NeuroImage.

[21] Peters B.D., Ikuta T., DeRosse P., John M., Burdick K.E., Gruner P., Prendergast D.M., Szeszko P.R., Malhotra A.K. (2014). Age-related differences in white matter tract microstructure are associated with cognitive performance from childhood to adulthood. Biol Psychiatry.

[22] Liston C., Watts R., Tottenham N., Davidson M.C., Niogi S., Ulug A.M., Casey B.J. (2006). Frontostriatal microstructure modulates efficient recruitment of cognitive control. Cereb Cortex N Y N 1991.

[23] Bos W., van den, Rodriguez C.A., Schweitzer J.B., McClure S.M. (2015). Adolescent impatience decreases with increased frontostriatal connectivity. Proc Natl Acad Sci U S A.

[24] Darki F., Klingberg T. (2015). The role of fronto-parietal and fronto-striatal networks in the development of working memory: a longitudinal study. Cereb Cortex.

[25] Marner L., Nyengaard J.R., Tang Y., Pakkenberg B. (2003). Marked loss of myelinated nerve fibers in the human brain with age. J Comp Neurol.

[26] Peters A. (2002). The effects of normal aging on myelin and nerve fibers: a review. J Neurocytol.

[27] Cox S.R., Ritchie S.J., Tucker-Drob E.M., Liewald D.C., Hagenaars S.P., Davies G., Wardlaw J.M., Gale C.R., Bastin M.E., Deary I.J. (2016). Ageing and brain white matter structure in 3,513 UK Biobank participants. Nat Commun.

[28] Douaud G., Groves A.R., Tamnes C.K., Westlye L.T., Duff E.P., Engvig A., Walhovd K.B., James A., Gass A., Monsch A.U. (2014). A common brain network links development, aging, and vulnerability to disease. Proc Natl Acad Sci U S A.

[29] Bennett I.J., Madden D.J. (2014). Disconnected aging: cerebral white matter integrity and age-related differences in cognition. Neuroscience.

[30] Hofstetter S., Tavor I., Moryosef S.T., Assaf Y. (2013). Short-term learning induces white matter plasticity in the fornix. J Neurosci.

[31] Taubert M., Draganski B., Anwander A., Müller K., Horstmann A., Villringer A., Ragert P. (2010). Dynamic properties of human brain structure: learning-related changes in cortical areas and associated fiber connections. J Neurosci.

[32] Alonso-Ortiz E., Levesque I.R., Pike G.B. (2015). MRI-based myelin water imaging: a technical review. Magn Reson Med.

[33] Prasloski T., Rauscher A., MacKay A.L., Hodgson M., Vavasour I.M., Laule C., Mädler B. (2012). Rapid whole cerebrum myelin water imaging using a 3D GRASE sequence. NeuroImage.

[34] Blumenfeld-Katzir T., Pasternak O., Dagan M., Assaf Y. (2011). Diffusion MRI of structural brain plasticity induced by a learning and memory task. PLoS ONE.

[35] Yeung M.S.Y., Zdunek S., Bergmann O., Bernard S., Salehpour M., Alkass K., Perl S., Tisdale J., Possnert G., Brundin L. (2014). Dynamics of oligodendrocyte generation and myelination in the human brain. Cell.

[36] Young K.M., Psachoulia K., Tripathi R.B., Dunn S-J., Cossell L., Attwell D., Tohyama K., Richardson W.D. (2013). Oligodendrocyte dynamics in the healthy adult CNS: evidence for myelin remodeling. Neuron.

[37] Schneider S., Gruart A., Grade S., Zhang Y., Kröger S., Kirchhoff F., Eichele G., García D.M.J., Dimou L. (2016). Decrease in newly generated oligodendrocytes leads to motor dysfunctions and changed myelin structures that can be rescued by transplanted cells. Glia.

[38•] Jeffries M.A., Urbanek K., Torres L., Wendell S.G., Rubio M.E., Fyffe-Maricich S.L. (2016). ERK1/2 activation in preexisting oligodendrocytes of adult mice drives new myelin synthesis and enhanced CNS function. J Neurosci.

[39] Snaidero N., Möbius W., Czopka T., Hekking L.H.P., Mathisen C., Verkleij D., Goebbels S., Edgar J., Merkler D., Lyons D.A. (2014). Myelin membrane wrapping of CNS axons by PI(3,4,5)P3-dependent polarized growth at the inner tongue. Cell.

[40] Goebbels S., Oltrogge J.H., Kemper R., Heilmann I., Bormuth I., Wolfer S., Wichert S.P., Möbius W., Liu X., Lappe-Siefke C. (2010). Elevated phosphatidylinositol 3,4,5-trisphosphate in glia triggers cell-autonomous membrane wrapping and myelination. J Neurosci Off J Soc Neurosci.

[41] de Hoz L., Simons M. (2015). The emerging functions of oligodendrocytes in regulating neuronal network behaviour. BioEssays.

[42] Dan Y., Poo M-M. (2006). Spike timing-dependent plasticity: from synapse to perception. Physiol Rev.

[43] Pajevic S., Basser P.J., Fields R.D. (2014). Role of myelin plasticity in oscillations and synchrony of neuronal activity. Neuroscience.

[44•] Etxeberria A., Hokanson K.C., Dao D.Q., Mayoral S.R., Mei F., Redmond S.A., Ullian E.M., Chan J.R. (2016). Dynamic modulation of myelination in response to visual stimuli alters optic nerve conduction velocity. J Neurosci.

[45] Ford M.C., Alexandrova O., Cossell L., Stange-Marten A., Sinclair J., Kopp-Scheinpflug C., Pecka M., Attwell D., Grothe B. (2015). Tuning of Ranvier node and internode properties in myelinated axons to adjust action potential timing. Nat Commun.

[46•] Arancibia-Cárcamo I.L., Ford M.C., Cossell L., Ishida K., Tohyama K., Attwell D. (2017). Node of Ranvier length as a potential regulator of myelinated axon conduction speed. eLife.

[47] Chomiak T., Hu B. (2009). What is the optimal value of the *g*-ratio for myelinated fibers in the rat CNS? A theoretical approach. PLoS ONE.

[48] Walhovd K.B., Johansen-Berg H., Káradóttir R.T. (2014). Unraveling the secrets of white matter — bridging the gap between cellular, animal and human imaging studies. Neuroscience.

[49] Watkins T.A., Emery B., Mulinyawe S., Barres B.A. (2008). Distinct stages of myelination regulated by gamma-secretase and astrocytes in a rapidly myelinating CNS coculture system. Neuron.

[50] Czopka T., ffrench-Constant C., Lyons D.A. (2013). Individual oligodendrocytes have only a few hours in which to generate new myelin sheaths in vivo. Dev Cell.

[51] Freeman S.A., Desmazières A., Simonnet J., Gatta M., Pfeiffer F., Aigrot M.S., Rappeneau Q., Guerreiro S., Michel P.P., Yanagawa Y. (2015). Acceleration of conduction velocity linked to clustering of nodal components precedes myelination. Proc Natl Acad Sci U S A.

[52] Fünfschilling U., Supplie L.M., Mahad D., Boretius S., Saab A.S., Edgar J., Brinkmann B.G., Kassmann C.M., Tzvetanova I.D., Möbius W. (2012). Glycolytic oligodendrocytes maintain myelin and long-term axonal integrity. Nature.

[53] Liu J., Dietz K., DeLoyht J.M., Pedre X., Kelkar D., Kaur J., Vialou V., Lobo M.K., Dietz D.M., Nestler E.J. (2012). Impaired adult myelination in the prefrontal cortex of socially isolated mice. Nat Neurosci.

[54] Makinodan M., Rosen K.M., Ito S., Corfas G. (2012). A critical period for social experience-dependent oligodendrocyte maturation and myelination. Science.

[55] Marques S., Zeisel A., Codeluppi S., Bruggen D. van, Falcão A.M., Xiao L., Li H., Häring M., Hochgerner H., Romanov R.A. (2016). Oligodendrocyte heterogeneity in the mouse juvenile and adult central nervous system. Science.

[56] Tomassy G.S., Dershowitz L.B., Arlotta P. (2016). Diversity matters: a revised guide to myelination. Trends Cell Biol.

[57•] Bechler M.E., Byrne L., ffrench-Constant C. (2015). CNS myelin sheath lengths are an intrinsic property of oligodendrocytes. Curr Biol.

[58] Tomassy G.S., Berger D.R., Chen H-H., Kasthuri N., Hayworth K.J., Vercelli A., Seung H.S., Lichtman J.W., Arlotta P. (2014). Distinct profiles of myelin distribution along single axons of pyramidal neurons in the neocortex. Science.

[59•] Koudelka S., Voas M.G., Almeida R.G., Baraban M., Soetaert J., Meyer M.P., Talbot W.S., Lyons D.A. (2016). Individual neuronal subtypes exhibit diversity in CNS myelination mediated by synaptic vesicle release. Curr Biol.

[60] Kasthuri N., Hayworth K.J., Berger D.R., Schalek R.L., Conchello J.A., Knowles-Barley S., Lee D., Vázquez-Reina A., Kaynig V., Jones T.R. (2015). Saturated reconstruction of a volume of neocortex. Cell.

[61] Briggman K.L., Bock D.D. (2012). Volume electron microscopy for neuronal circuit reconstruction. Curr Opin Neurobiol.

[62] Stüber C., Morawski M., Schäfer A., Labadie C., Wähnert M., Leuze C., Streicher M., Barapatre N., Reimann K., Geyer S. (2014). Myelin and iron concentration in the human brain: a quantitative study of MRI contrast. NeuroImage.

[63] Chen W.C., Foxley S., Miller K.L. (2013). Detecting microstructural properties of white matter based on compartmentalization of magnetic susceptibility. NeuroImage.

